# *catena*-Poly[aqua­bis­(μ-3-chloro­benzo­ato-κ^2^ *O*:*O*′)zinc]

**DOI:** 10.1107/S160053681301564X

**Published:** 2013-06-15

**Authors:** Nihat Bozkurt, Nefise Dilek, Nagihan Çaylak Delibaş, Hacali Necefoğlu, Tuncer Hökelek

**Affiliations:** a Department of Chemistry, Kafkas University, 36100 Kars, Turkey; b Aksaray University, Department of Physics, 68100, Aksaray, Turkey; c Department of Physics, Sakarya University, 54187 Esentepe, Sakarya, Turkey; d Department of Physics, Hacettepe University, 06800 Beytepe, Ankara, Turkey

## Abstract

In the polymeric title compound, [Zn(C_7_H_4_ClO_2_)_2_(H_2_O)]_*n*_, the Zn^II^ cation is located on a twofold rotation axis and is coordinated by carboxylate O atoms of four monodentate chloro­benzoate anions and by one water mol­ecule, located on a twofold rotation axis, in a distorted square-pyramidal geometry. In the anion, the carboxyl­ate group is twisted away from the attached benzene ring by 44.16 (11)°. The chloro­benzoate anion bridges Zn^II^ cations, forming polymeric chains running along the *c-*axis direction. O—H⋯O hydrogen bonds between coordinating water mol­ecules and carboxyl­ate groups link adjacent chains into layers parallel to the *bc* plane.

## Related literature

For structural functions and coordination relationships of the aryl­carboxyl­ate ion in transition metal complexes of benzoic acid derivatives, see: Nadzhafov *et al.* (1981 ▶); Shnulin *et al.* (1981 ▶). For applications of transition metal complexes with biochemical mol­ecules in biological systems, see: Antolini *et al.* (1982 ▶). Some benzoic acid derivatives, such as 4-amino­benzoic acid, have been extensively reported in coordination chemistry, as bifunctional organic ligands, due to the varieties of their coordination modes, see: Chen & Chen (2002 ▶); Amiraslanov *et al.* (1979 ▶); Hauptmann *et al.* (2000 ▶). For related structures, see: Aydın *et al.* (2012 ▶); Hökelek *et al.* (2009 ▶, 2010*a* ▶,*b* ▶, 2011 ▶); Necefoğlu *et al.* (2011 ▶); Zaman *et al.* (2012 ▶). For bond-length data, see: Allen *et al.* (1987 ▶).
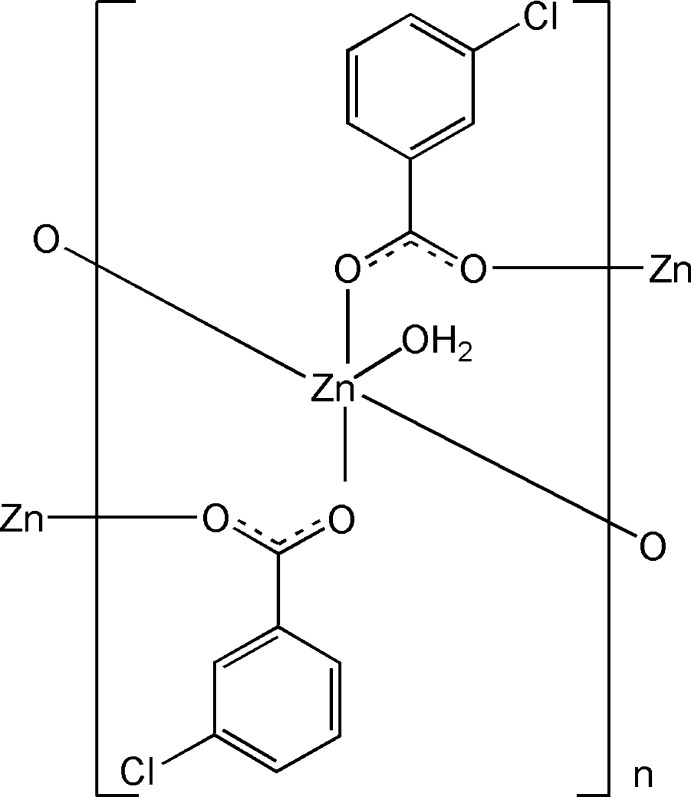


## Experimental

### 

#### Crystal data

[Zn(C_7_H_4_ClO_2_)_2_(H_2_O)]
*M* *_r_* = 394.51
Monoclinic, 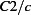

*a* = 31.8553 (8) Å
*b* = 6.1786 (2) Å
*c* = 7.5117 (3) Å
β = 96.554 (2)°
*V* = 1468.80 (8) Å^3^
*Z* = 4
Mo *K*α radiation
μ = 2.06 mm^−1^
*T* = 294 K
0.35 × 0.25 × 0.15 mm

#### Data collection

Bruker SMART BREEZE CCD diffractometer
Absorption correction: multi-scan (*SADABS*; Bruker, 2012 ▶) *T*_min_ = 0.545, *T*_max_ = 0.735
13582 measured reflections
1825 independent reflections
1727 reflections with *I* > 2σ(*I*)
*R*_int_ = 0.036

#### Refinement

*R*[*F* ^2^ > 2σ(*F* ^2^)] = 0.026
*wR*(*F* ^2^) = 0.069
*S* = 1.12
1825 reflections
105 parameters
H atoms treated by a mixture of independent and constrained refinement
Δρ_max_ = 0.43 e Å^−3^
Δρ_min_ = −0.35 e Å^−3^

### 

Data collection: *APEX2* (Bruker, 2012 ▶); cell refinement: *SAINT* (Bruker, 2012 ▶); data reduction: *SAINT*; program(s) used to solve structure: *SHELXS97* (Sheldrick, 2008 ▶); program(s) used to refine structure: *SHELXL97* (Sheldrick, 2008 ▶); molecular graphics: *ORTEP-3* for Windows (Farrugia, 2012 ▶); software used to prepare material for publication: *WinGX* (Farrugia, 2012 ▶) and *PLATON* (Spek, 2009 ▶).

## Supplementary Material

Crystal structure: contains datablock(s) I, global. DOI: 10.1107/S160053681301564X/xu5711sup1.cif

Structure factors: contains datablock(s) I. DOI: 10.1107/S160053681301564X/xu5711Isup2.hkl

Additional supplementary materials:  crystallographic information; 3D view; checkCIF report

## Figures and Tables

**Table 1:** Selected bond lengths (Å)

Zn1—O1	2.1779 (12)
Zn1—O2	1.9493 (11)
Zn1—O3	1.9664 (19)

**Table 2:** Hydrogen-bond geometry (Å, °)

*D*—H⋯*A*	*D*—H	H⋯*A*	*D*⋯*A*	*D*—H⋯*A*
O3—H31⋯O1^i^	0.77 (2)	1.89 (2)	2.6421 (17)	168 (2)

Symmetry code: (i) 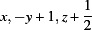.

**Fig. 1.**
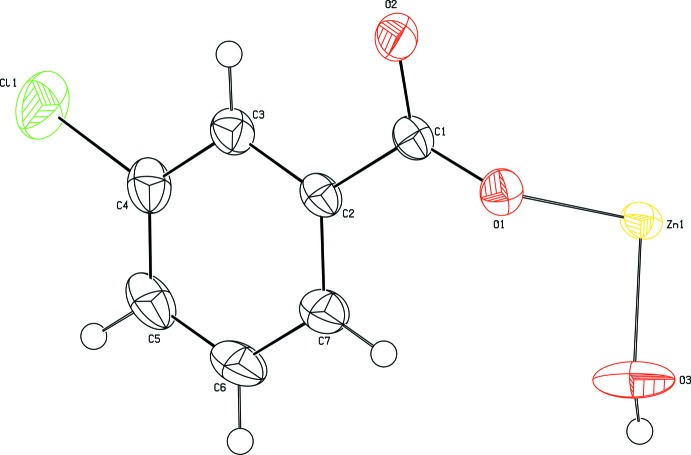
The asymmetric unit of the title molecule with the atom-numbering scheme. Displacement ellipsoids are drawn at the 50% probability level.

**Fig. 2.**
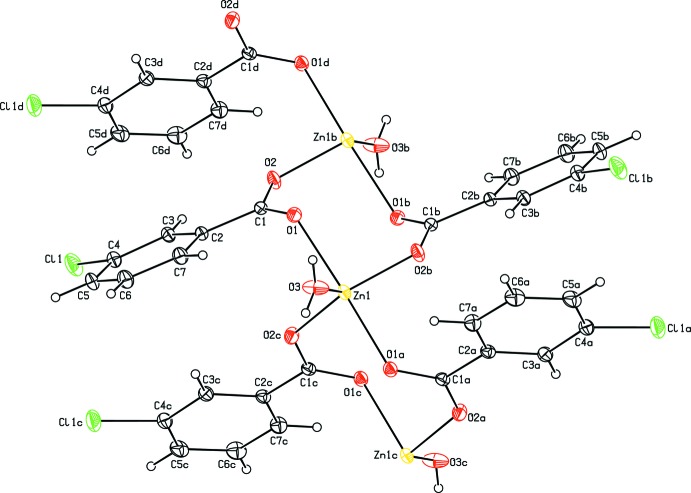
Part of the polymeric chain of the title compound.

**Fig. 3.**
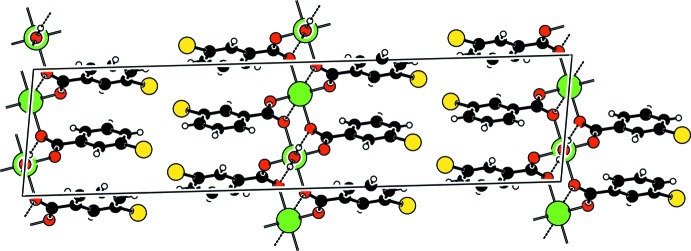
A view along the b axis of the packing of the title compound (a axis horizontal; c axis vertical). Hydrogen bonds are shown as dashed lines.

## supplementary crystallographic information

### Comment

The structural functions and coordination relationships of the arylcarboxylate ion in transition metal complexes of benzoic acid derivatives change depending on the nature and position of the substituent groups on the benzene ring, the nature of the additional ligand molecule or solvent, and the medium of the synthesis (Nadzhafov *et al.*, 1981; Shnulin *et al.*, 1981). Transition metal complexes with biochemically active ligands frequently show interesting physical and/or chemical properties, as a result they may find applications in biological systems (Antolini *et al.*, 1982). Some benzoic acid derivatives, such as 4-aminobenzoic acid, have been extensively reported in coordination chemistry, as bifunctional organic ligands, due to the varieties of their coordination modes (Chen & Chen, 2002; Amiraslanov *et al.*, 1979; Hauptmann *et al.*, 2000). The title compound was synthesized and its crystal structure is reported herein.

The asymmetric unit of the title compound, (I), contains one-half Zn^II^ cation, one chlorobenzoate (CB) anion and one-half water molecule (Fig. 1). In the crystal, two CB anions bridge adjacent Zn^II^ cations, forming a polymeric chain running along the *c* axis, while the water molecule coordinate in a monodentate manner to the Zn^II^ cation, completing the distorted square-pyramidal geometry (Fig. 2). As a result of the CB anions bridging of the adjacent Zn^II^ cations, an eight-membered ring is formed where the distances between the symmetry related atoms, Zn1···Zn1b [4.3798 (3) Å], O1···O1b [3.020 (2) Å], O2···O2b [4.337 (2) Å] and C1···C1b [3.975 (2) Å] [symmetry code: (b) - *x*, - *y*, 1 - *z*], may reflect its size.

The crystal structures of some benzoate containing polymeric complexes of Mn^II^, Zn^II^, Pb^II^ and Co^II^ ions, [Mn_2_(C_8_H_7_O_2_)_4_(C_10_H_14_N_2_O)_2_(H_2_O)]_n_ (Hökelek *et al.*, 2010*a*), [Mn(C_7_H_4_FO_2_)_2_(H_2_O)_2_]_n_ (Necefoğlu *et al.*, 2011), [Zn(C_8_H_5_O_3_)_2_(C_6_H_6_N_2_O)]_n_ (Hökelek *et al.*, 2009), [Pb(C_8_H_7_O_2_)_2_(C_6_H_6_N_2_O)]_n_ (Hökelek *et al.*, 2010*b*), {[Pb(C_9_H_9_O_2_)_2_(C_6_H_6_N_2_O)].H_2_O}_n_ (Hökelek *et al.*, 2011), {[Pb(C_7_H_5_O_3_)_2_(C_6_H_6_N_2_O)].H_2_O}_n_ (Zaman *et al.*, 2012) and [Co(C_7_H_4_IO_2_)_2_(H_2_O)_2_]_n_ (Aydın *et al.*, 2012) have also been reported.

In the title compound, the four O atoms (O1, O1a, O2b and O2c) [symmetry codes: (a) - *x*, *y*, 1/2 - *z*, (b) - *x*, - *y*, 1 - *z*, (c) *x*, - *y*, - 1/2 + *z*] in the equatorial plane around the Zn^II^ cation form a distorted square-planar arrangement, while the distorted square-pyramidal geometry is completed by the water O atom (O3) in the axial position. The near equalities of the C1—O1 [1.260 (2) Å] and C1—O2 [1.258 (2) Å] bonds in the carboxylate group indicate delocalized bonding arrangement, rather than localized single and double bonds. The average Zn—O bond length is 2.0636 (12) Å (for benzoate oxygens) and 1.9664 (19) Å (for water oxygen) (Table 1) close to standard values (Allen *et al.*, 1987). The Zn atom is displaced out of the mean-plane of the carboxylate group (O1/C1/O2) by 1.3998 (1) Å. Atoms Cl1, C1 and O1 are -0.0897 (7), -0.0181 (16) and -0.2341 (12) Å away from the mean-plane of the adjacent benzene ring, respectively. The dihedral angle between the planar carboxylate group (O1/C1/O2) and the adjacent benzene ring *A* (C2—C7) is 44.16 (11)°.

In the crystal, strong O—H···O hydrogen bonds (Table 2) link the water hydrogens to the carboxylate oxygens in the polymeric chains (Fig. 3).

### Experimental

The title compound was prepared by the reaction of ZnSO_4_.H_2_O (0.89 g, 5 mmol) in H_2_O (50 ml) with sodium 3-chlorobenzoate (1.79 g, 10 mmol) in H_2_O (100 ml) at room temperature. The mixture was filtered and set aside to crystallize at ambient temperature for one week, giving colorless single crystals.

### Refinement

Atom H31 (for H_2_O) was located in a difference Fourier map and was refined freely. The C-bound H-atoms were positioned geometrically with C—H = 0.93 Å for aromatic H-atoms, and constrained to ride on their parent atoms, with *U*_iso_(H) = 1.2*U*_eq_(C).

### Crystal data

**Table:**

[Zn(C_7_H_4_ClO_2_)_2_(H_2_O)]	*F*(000) = 792
*M**_r_* = 394.51	*D*_x_ = 1.784 Mg m^−^^3^
Monoclinic, *C*2/*c*	Mo *K*α radiation, λ = 0.71073 Å
Hall symbol: -C 2yc	Cell parameters from 9983 reflections
*a* = 31.8553 (8) Å	θ = 2.6–28.3°
*b* = 6.1786 (2) Å	µ = 2.06 mm^−^^1^
*c* = 7.5117 (3) Å	*T* = 294 K
β = 96.554 (2)°	Block, colorless
*V* = 1468.80 (8) Å^3^	0.35 × 0.25 × 0.15 mm
*Z* = 4	

### Data collection

**Table:**

Bruker SMART BREEZE CCD diffractometer	1825 independent reflections
Radiation source: fine-focus sealed tube	1727 reflections with *I* > 2σ(*I*)
Graphite monochromator	*R*_int_ = 0.036
φ and ω scans	θ_max_ = 28.3°, θ_min_ = 1.3°
Absorption correction: multi-scan (*SADABS*; Bruker, 2012)	*h* = −41→42
*T*_min_ = 0.545, *T*_max_ = 0.735	*k* = −8→8
13582 measured reflections	*l* = −8→10

### Refinement

**Table:**

Refinement on *F*^2^	Primary atom site location: structure-invariant direct methods
Least-squares matrix: full	Secondary atom site location: difference Fourier map
*R*[*F*^2^ > 2σ(*F*^2^)] = 0.026	Hydrogen site location: inferred from neighbouring sites
*wR*(*F*^2^) = 0.069	H atoms treated by a mixture of independent and constrained refinement
*S* = 1.12	*w* = 1/[σ^2^(*F*_o_^2^) + (0.0337*P*)^2^ + 1.4314*P*] where *P* = (*F*_o_^2^ + 2*F*_c_^2^)/3
1825 reflections	(Δ/σ)_max_ = 0.002
105 parameters	Δρ_max_ = 0.43 e Å^−^^3^
0 restraints	Δρ_min_ = −0.35 e Å^−^^3^

### Special details

**Table:**

Geometry. All esds (except the esd in the dihedral angle between two l.s. planes) are estimated using the full covariance matrix. The cell esds are taken into account individually in the estimation of esds in distances, angles and torsion angles; correlations between esds in cell parameters are only used when they are defined by crystal symmetry. An approximate (isotropic) treatment of cell esds is used for estimating esds involving l.s. planes.
Refinement. Refinement of F^2^ against ALL reflections. The weighted R-factor wR and goodness of fit S are based on F^2^, conventional R-factors R are based on F, with F set to zero for negative F^2^. The threshold expression of F^2^ > 2sigma(F^2^) is used only for calculating R-factors(gt) etc. and is not relevant to the choice of reflections for refinement. R-factors based on F^2^ are statistically about twice as large as those based on F, and R- factors based on ALL data will be even larger.

### Fractional atomic coordinates and isotropic or equivalent isotropic displacement parameters (Å^2^)

**Table:**

	*x*	*y*	*z*	*U*_iso_*/*U*_eq_	
Zn1	1.0000	0.18233 (4)	0.2500	0.02388 (10)	
Cl1	0.781898 (15)	−0.05564 (11)	−0.15745 (9)	0.05827 (18)	
O1	0.97446 (4)	0.20515 (18)	−0.03094 (16)	0.0286 (3)	
O2	1.05402 (4)	0.0674 (2)	0.19485 (18)	0.0368 (3)	
O3	1.0000	0.5006 (3)	0.2500	0.0497 (6)	
H31	0.9935 (8)	0.573 (4)	0.325 (3)	0.045 (7)*	
C1	0.94299 (5)	0.1000 (3)	−0.1019 (2)	0.0250 (3)	
C2	0.89932 (5)	0.1772 (2)	−0.0796 (2)	0.0263 (3)	
C3	0.86489 (5)	0.0444 (3)	−0.1307 (2)	0.0306 (3)	
H3	0.8687	−0.0906	−0.1814	0.037*	
C4	0.82482 (5)	0.1162 (3)	−0.1050 (3)	0.0357 (4)	
C5	0.81842 (6)	0.3189 (3)	−0.0349 (3)	0.0409 (5)	
H5	0.7913	0.3660	−0.0205	0.049*	
C6	0.85288 (6)	0.4504 (3)	0.0133 (3)	0.0411 (4)	
H6	0.8489	0.5872	0.0600	0.049*	
C7	0.89340 (6)	0.3808 (3)	−0.0069 (3)	0.0346 (4)	
H7	0.9165	0.4694	0.0277	0.042*	

### Atomic displacement parameters (Å^2^)

**Table:**

	*U*^11^	*U*^22^	*U*^33^	*U*^12^	*U*^13^	*U*^23^
Zn1	0.02038 (13)	0.01944 (13)	0.03266 (17)	0.000	0.00670 (10)	0.000
Cl1	0.0262 (2)	0.0787 (4)	0.0707 (4)	−0.0071 (2)	0.0089 (2)	−0.0203 (3)
O1	0.0262 (5)	0.0295 (6)	0.0302 (6)	−0.0027 (4)	0.0034 (5)	0.0041 (4)
O2	0.0225 (5)	0.0460 (7)	0.0416 (7)	0.0066 (5)	0.0027 (5)	−0.0187 (6)
O3	0.0973 (18)	0.0189 (8)	0.0384 (12)	0.000	0.0312 (12)	0.000
C1	0.0237 (7)	0.0287 (7)	0.0231 (8)	0.0039 (6)	0.0049 (6)	0.0027 (6)
C2	0.0241 (7)	0.0296 (8)	0.0256 (8)	0.0060 (5)	0.0052 (6)	0.0013 (6)
C3	0.0256 (7)	0.0356 (8)	0.0310 (9)	0.0043 (6)	0.0056 (6)	−0.0034 (7)
C4	0.0249 (8)	0.0485 (10)	0.0340 (9)	0.0030 (7)	0.0046 (7)	−0.0012 (8)
C5	0.0302 (9)	0.0523 (12)	0.0418 (11)	0.0169 (8)	0.0105 (8)	0.0013 (8)
C6	0.0432 (10)	0.0355 (9)	0.0459 (11)	0.0151 (8)	0.0107 (8)	−0.0036 (8)
C7	0.0337 (8)	0.0311 (8)	0.0395 (10)	0.0049 (7)	0.0062 (7)	−0.0030 (7)

### Geometric parameters (Å, º)

**Table:**

Zn1—O1	2.1779 (12)	C2—C3	1.388 (2)
Zn1—O1^i^	2.1779 (12)	C2—C7	1.393 (2)
Zn1—O2	1.9493 (11)	C3—C4	1.386 (2)
Zn1—O2^i^	1.9493 (11)	C3—H3	0.9300
Zn1—O3	1.9664 (19)	C5—C4	1.383 (3)
Cl1—C4	1.740 (2)	C5—C6	1.380 (3)
O1—C1	1.260 (2)	C5—H5	0.9300
O2—C1^ii^	1.258 (2)	C6—H6	0.9300
O3—H31	0.77 (2)	C7—C6	1.385 (2)
C1—O2^ii^	1.258 (2)	C7—H7	0.9300
C2—C1	1.498 (2)		
			
O1—Zn1—O1^i^	172.58 (6)	C3—C2—C7	120.29 (15)
O2—Zn1—O1	93.38 (5)	C7—C2—C1	120.01 (15)
O2^i^—Zn1—O1	89.33 (5)	C2—C3—H3	120.5
O2—Zn1—O1^i^	89.33 (5)	C4—C3—C2	118.90 (16)
O2^i^—Zn1—O1^i^	93.38 (5)	C4—C3—H3	120.5
O2^i^—Zn1—O2	137.26 (8)	C3—C4—Cl1	119.05 (16)
O2—Zn1—O3	111.37 (4)	C5—C4—Cl1	119.50 (14)
O2^i^—Zn1—O3	111.37 (4)	C5—C4—C3	121.43 (18)
O3—Zn1—O1	86.29 (3)	C4—C5—H5	120.5
O3—Zn1—O1^i^	86.29 (3)	C6—C5—C4	119.05 (16)
C1—O1—Zn1	124.58 (10)	C6—C5—H5	120.5
C1^ii^—O2—Zn1	122.84 (11)	C5—C6—C7	120.76 (17)
Zn1—O3—H31	125.7 (19)	C5—C6—H6	119.6
O1—C1—C2	119.52 (14)	C7—C6—H6	119.6
O2^ii^—C1—O1	123.47 (14)	C2—C7—H7	120.2
O2^ii^—C1—C2	117.00 (14)	C6—C7—C2	119.54 (18)
C3—C2—C1	119.70 (14)	C6—C7—H7	120.2
			
O2—Zn1—O1—C1	116.58 (13)	C7—C2—C1—O2^ii^	−168.37 (16)
O2^i^—Zn1—O1—C1	−20.74 (13)	C1—C2—C3—C4	178.42 (16)
O3—Zn1—O1—C1	−132.21 (12)	C7—C2—C3—C4	−1.2 (3)
O1—Zn1—O2—C1^ii^	−55.67 (14)	C1—C2—C7—C6	−179.91 (17)
O1^i^—Zn1—O2—C1^ii^	131.24 (14)	C3—C2—C7—C6	−0.3 (3)
O2^i^—Zn1—O2—C1^ii^	36.99 (13)	C2—C3—C4—Cl1	−176.52 (14)
O3—Zn1—O2—C1^ii^	−143.01 (13)	C2—C3—C4—C5	2.0 (3)
Zn1—O1—C1—O2^ii^	−100.74 (17)	C6—C5—C4—Cl1	177.28 (16)
Zn1—O1—C1—C2	80.48 (17)	C6—C5—C4—C3	−1.2 (3)
C3—C2—C1—O1	−169.14 (15)	C4—C5—C6—C7	−0.3 (3)
C3—C2—C1—O2^ii^	12.0 (2)	C2—C7—C6—C5	1.1 (3)
C7—C2—C1—O1	10.5 (2)		

Symmetry codes: (i) −*x*+2, *y*, −*z*+1/2; (ii) −*x*+2, −*y*, −*z*.

### Hydrogen-bond geometry (Å, º)

**Table:**

*D*—H···*A*	*D*—H	H···*A*	*D*···*A*	*D*—H···*A*
O3—H31···O1^iii^	0.77 (2)	1.89 (2)	2.6421 (17)	168 (2)

Symmetry code: (iii) *x*, −*y*+1, *z*+1/2.
